# Length of stay following vaginal deliveries: A population based study in the Friuli Venezia Giulia region (North-Eastern Italy), 2005-2015

**DOI:** 10.1371/journal.pone.0204919

**Published:** 2019-01-03

**Authors:** Luca Cegolon, Oona Campbell, Salvatore Alberico, Marcella Montico, Giuseppe Mastrangelo, Lorenzo Monasta, Luca Ronfani, Fabio Barbone

**Affiliations:** 1 Institute for Maternal and Child Health, IRCCS “Burlo Garofolo”, Scientific Directorate, Trieste, Italy; 2 London School of Hygiene & Tropical Medicine, MARCH Centre, Faculty of Epidemiology & Population Health, London, United Kingdom; 3 Institute for Maternal and Child Health, IRCCS “Burlo Garofolo”, Clinical Epidemiology & Public Health Research Unit, Trieste, Italy; 4 Padua University, Department of Cardio-Thoracic & Vascular Sciences, Padua, Italy; Centre Hospitalier Universitaire Vaudois, FRANCE

## Abstract

**Background:**

Lengths of hospital stay (LoS) after childbirth that are too long have a number of health, social and economic drawbacks. For this reason, in several high-income countries LoS has been reduced over the past decades and early discharge (ED) is increasingly applied to low-risk mothers and newborns.

**Methods:**

We conducted a population-based study investigating LoS after chilbirth across all 12 maternity centres of Friuli Venezia-Giulia (FVG), North-Eastern Italy, using a database capturing all registered births in the region from 2005 to 2015 (11 years). Adjusting for clinical factors (clinical conditions of the mother and the newborn), socio-demographic bakground and obstetric history with multivariable logistic regression, we ranked facility centres for LoS that were longer than our proposed ED benchmarks (defined as >2 days for spontaneous vaginal deliveries and >3 days for instrumental vaginal deliveries). The reference was hospital A, a national excellence centre for maternal and child health.

**Results:**

The total number of births examined in our database was 109,550, of which 109,257 occurred in hospitals. During these 11 years, the number of births significantly diminished over time, and the pooled mean LoS for spontaneous vaginal deliveries in the whole FVG was 2.9 days. There was a significantly decreasing trend in the proportion of women remaining admitted more than the respective ED cutoffs for both delivery modes. The percentage of women staying longer that the ED benchmarks varied extensively by facility centre, ranging from 32% to 97% for spontaneous vaginal deliveries and 15% to 64% for instrumental vaginal deliveries. All hospitals but G were by far more likely to surpass the ED cutoff for spontaneous deliveries. As compared with hospital A, the most significant adjusted ORs for LoS overcoming the ED thresholds for spontaneous vaginal deliveries were: 89.38 (78.49–101.78); 26.47 (22.35–31.36); 10.42 (9.49–11.44); 10.30 (9.45–11.21) and 8.40 (7.68–9.19) for centres B, D, I, K and E respectively. By contrast the OR was 0.77 (95%CI: 0.72–0.83) for centre G. Similar mitigated patterns were observed also for instrumental vaginal deliveiries.

**Conclusions:**

For spontaneous vaginal deliveries the mean LoS in the whole FVG was shorter than 3.4 days, the average figure most recently reported for the whole of Italy, but higher than other countries’ with health systems similar to Italy’s. Since our results are controlled for the effect of all other factors, the between-hospital variability we found is likely attributable to the health care provider itself. It can be argued that some maternity centres of FVG may have had ecocomic interest in longer LoS after childbirth, although fear of medico-legal backlashes, internal organizational malfunctions of hospitals and scarce attention of ward staff on performance efficiency shall not be ruled out. It would be therefore important to ensure higher level of coordination between the various maternity services of FVG, which should follow standardized protocols to pursue efficiency of care and allow comparability of health outcomes and costs among them. Improving the performance of FVG and Italian hospitals requires investment in primary care services.

## Background

Albeit birth is a wellness and natural event (not an illness), almost all babies in high-middle income countries are delivered in hospital, where postnatal care is provided with the goal of monitoring and treating the mother and the newborn for eventual complications. Hospital postnatal care includes also necessary support to the woman for her transition home, counseling on breastfeeding and health promotion indications [1].

There is no consensus around appropriate length of hospital stay (LoS) after childbirth; the only recommendation (based on weak evidence) is from the World Health Organization (WHO), according to which all women should remain admitted at least 24h postpartum [2]. This recommendation aims to ensure that the mother and the newborn, particularly in low-income countries, remain long enough in hospital to be appropriately monitored by skilled birth attendants, in the event of serious complications requiring emergency care. The first 24h post-partum in fact poses the greatest risk of fatal events (often not predictable) both for the mother and the newborn [3].

However, in high-income countries the perspective is almost overturned, as hospitalization after childbirth may also present some downsides. For instance, hospital postnatal stays that are too long may expose the mother and the newborn to risk of nosocomial infections, which increases with LoS [4]. Extended LoS could also have an impact on family ties, as the partner is often involved and sibling competition could be triggered [4]. Longer LoS may also cause in the mother sleeping disorders, stress, breastfeeding issues and dissatisfaction toward the health care service received [5,6]. Finally, LoS that are long can also have a negative economic impact on hospital performance and sustainability of health systems, especially those funded by central taxation [7]. Therefore, to contain unnecessary days of LoS, postnatal care has been going through considerable evolution in the Western World over the past 30 years [8].

In particular, the average LoS after childbirth has been progressively shortened to improve patient satisfaction and reduce health care costs associated with childbirth. Concepts such as early discharge (ED) have taken place in several high-income countries, where the average LoS for spontaeous vaaginal deliveries (SVD) is now 48 hours or less [4,9] and discharge even within a few hours after birth is not uncommon nowadays [10]. Despite an increase in medical interventions during childbirth and more complex needs of pregnant women, there is in fact evidence that in several high-income countries low-risk mothers and newborn are being discharged as early as 4–6 h after childbirth [11–13]. For instance (although it may be a special case) the Duchess of Cambridge of the UK was reportedly been discharged from S. Mary’s Hospital in London 24h, 10h and 7h after delivering her three “royal babies” respectively [14].

There is no standardized definition of ED, as what is considered ED in one country may not be considered ED in another [15–17]. However, the most shared approach comes from the American Academy of Pediatrics and the American College of Obstetricians and Gynecologists, which in 1992 defined early postnatal discharge as a LoS less than 48h for women who had SVD and less than 96h for those who delivered by cesarean section (CS) [18].

There is considerable variability of LoS across the globe and ED is not applied systematically in all high-income countries, as a reflection not only of cultural and political diversity, but also difference in health systems [16]. Variability in LoS is also observed within Europe, with post-communist Eastern European countries reporting average LoS of 5–6 days for SVD, which considerably contrasts with figures from Western European countries. The former reports are likely the aftermath of previous communist health care policies still continuing nowadays [4,19]. However, even within Western Europe the situation is far from uniform. Whilst central European countries with Bismarckian health systems based upon social insurances (France, Luxembourg, Austria, Belgium, etc.) reported average LoS of around 4 days for SVD, in countries characterized by Beveridgean health systems founded upon central taxation (UK, Ireland, Netherlands, Sweden, Italy, etc.) the average LoS was about 2 days or even less [4,19,20]. Moreover, even within Beveridgean health systems, countries like Greece and Italy had an average LoS for SVD of 4.0 and 3.4 days respectively, which questions the performance and efficiency of the respective health systems [4,19].

A number of factors are reported to influence LoS according to the open literature: delivery mode, type of birth attendant, maternal age, parity, gestational age, birth-weight, multiple birth, infant survival status, analgesia, labour induction, maternal smoking, maternal body mass index (BMI), marital status, parents’ nationality, parental employment status, and others [4,8,17].

In view of the above, since ED is not applied in Italy nor in other European countries, we conducted a population based study in Friuli Venezia Giulia (FVG), a region of North-Eastern Italy, describing LoS after vaginal births and associated factors from 2005 to 2015, comparing the performance of the various FVG maternity centres to inform policy makers about the potential determinants of ED. We used a database capturing all registered births in the region during these 11 years to examine LoS by SVD as well as instrumental vaginal deliveries (IVD). Observations related to CS will be presented in another study.

## Methods

This study employed a cross-sectional design to investigate LoS and associated factors from 2005–2015 in FVG, a region of North-Eastern Italy with an approximately 1.22 million resident population, of which roughly 50% are females [21]. FVG has one of the most advanced information health system in Italy and has been historically at the forefront of health innovation in central Europe, so it is an ideal setting to experiment and evaluate potential new health care policies [22, 23].

### The database

**Fig 1:**
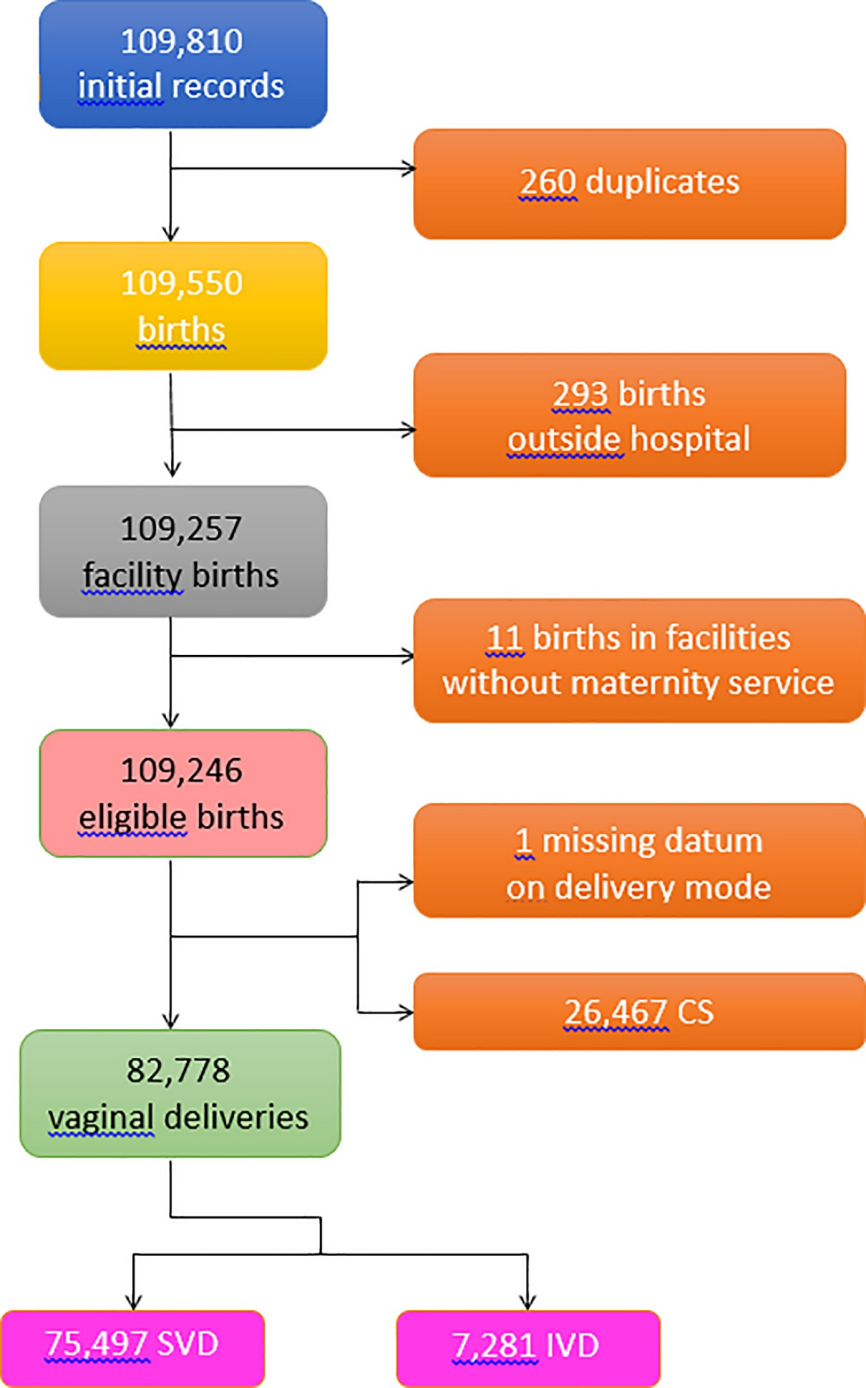
Flowchart displaying the various criteria applied to the initial database to obtain the final number of hospital records available for the analysis.

Data analyzed in the present study were extracted from the Regional Repository of FVG, a database anonymously storing administrative data from the Italian National Health Service (NHS) [21]. Fig 1 shows the flowchart displaying the various criteria applied to the initial database to obtain the final number of hospital records available for the analysis.

For our study we used the information collected by the Certificate of Delivery Care (CEDAP, Italian acronym) from all 12 hospitals with maternity services of FVG during 2005–2015. CEDAP is a formatted questionnaire filled up by trained health care personnel collecting clinical and personal information on women and newborn. Copy of CEDAP can be seen as a supplementary file (S1 Fig). The 12 regional maternity centres were anonymized and named by alphabetic letter from A to L.

Approval to conduct the study was granted by the Regional Health Authority of Friuli Venezia Giulia.

### Length of stay

LoS (measured in days) was calculated by subtracting the date of birth from the date of hospital discharge.

### Conceptual framework

**Fig 2:**
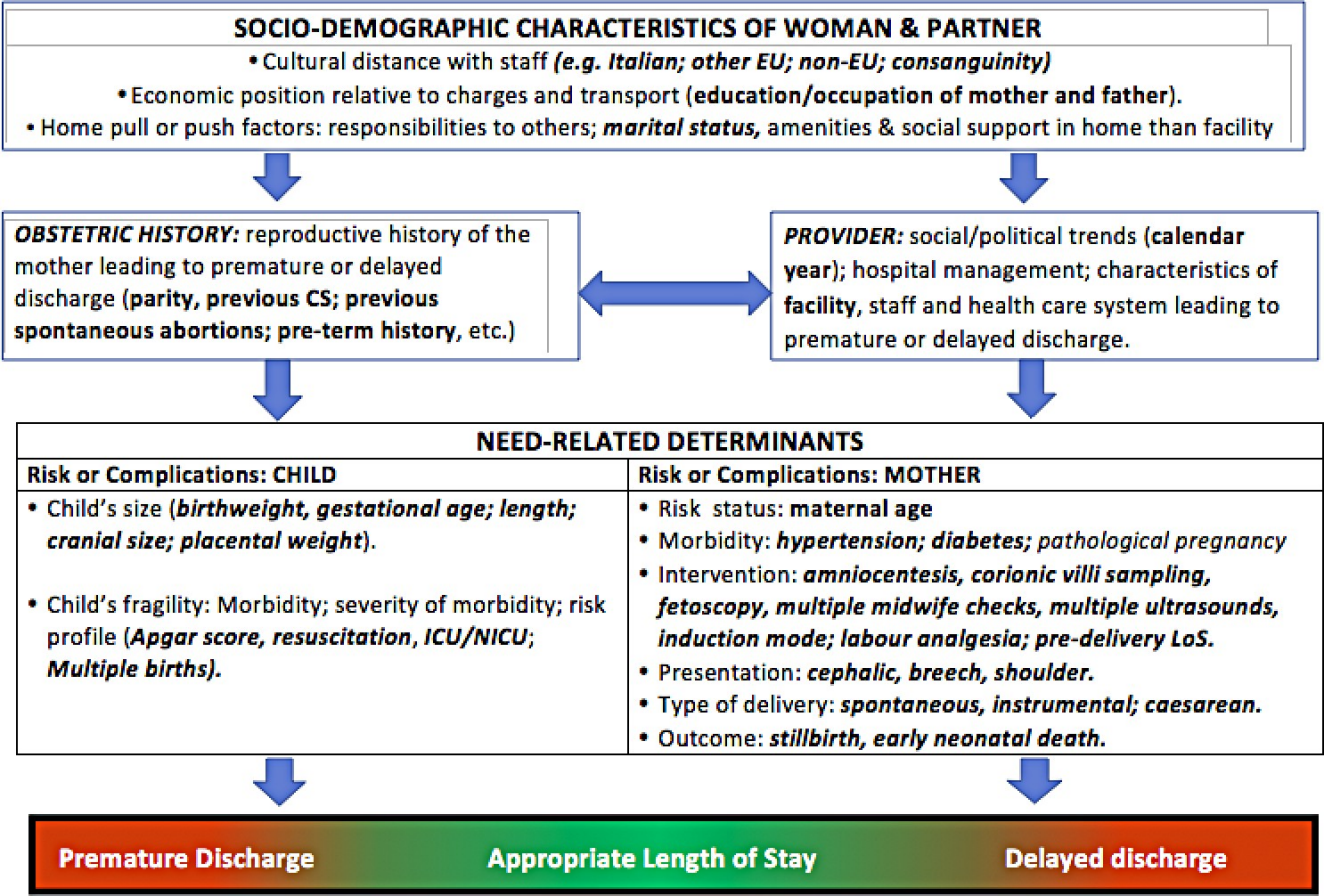
Conceptual framework explaining the relationship between various determinants and LoS.

We devised a conceptual framework combining our knowledge, the existing literature, and our reasoning to disentangle the relationships between various explanatory factors and LoS [24–26]. Conceptual frameworks lay out factors and concept domains as well as construct presumed relationships between determinants [24–26]. A previous model used four categories to describe determinants of LoS [27]: patient; healthcare providers; social/family environment; healthcare system. Our conceptual framework identified 5 broad domains of potential determinants of LoS (Fig 2):

1. Health care setting and calendar year;
2. Maternal health factors;
3. Clinical factors of the child:
  3.1 Child’s size;
  3.2 Child’s fragility;
4. Socio-demographic background;
5. Obstetric history.

### Variables

The following factors were used as explanatory variables in the analysis.

#### Health care setting

Table 1 shows the setting (hospitals) and the timeframe (calendar year) of the present investigation.

**Table 1:** Distibution of length of stay (LoS) after childbirth by healthcare setting and calendar year. Number; mean LoS (M) ± standard deviation (SD); row percentage (row %). NA = Not applicable.

FACTORS	STRATA	ALL BIRTHS		VAGINAL DELIVERY MODE															
				SPONTANEOUS (N = 75,497)								INSTRUMENTAL (N = 7,281)							
		Number	M ± SD (days)	LoS (days)								LoS (days)							
				M ± SD	≤1	2	3	4	5	6+	>2	M ± SD	≤1	2	3	4	5	6+	>3
					Row %								Row %						
**Calendar** **Year**	**2005**	10,177	3.5 ± 1.5	2.9 ±1.0	1.7	31.0	50.0	11.9	3.3	2.2	67.3	3.6 ± 1.7	0.9	16.0	43.4	23.6	8.0	8.2	39.8
	**2006**	10,470	3.4 ± 1.4	2.9 ±1.0	1.6	32.9	48.2	12.0	3.1	2.1	65.5	3.3 ± 1.1	0.3	17.6	48.0	23.6	6.1	4.5	34.2
	**2007**	10,652	3.4 ± 1.4	2.9 ± 1.0	1.0	34.6	48.3	11.8	2.7	1.6	64.4	3.4 ± 1.3	0.6	15.6	49.9	21.7	8.1	4.1	33.9
	**2008**	10,478	3.4 ± 1.4	2.9 ± 1.0	1.2	32.7	50.1	10.9	3.3	1.9	66.2	3.4 ± 1.2	0.6	14.7	48.2	23.1	7.5	5.9	36.5
	**2009**	10,492	3.4 ± 1.5	2.9 ± 1.2	1.3	33.0	49.0	11.5	3.0	2.2	65.7	3.3 ± 1.0	0.3	18.8	48.5	22.3	7.0	3.2	32.4
	**2010**	10,406	3.4 ± 1.4	2.9 ± 1.0	1.2	21.2	51.2	11.8	2.8	1.8	67.6	3.4 ± 1.3	0.4	15.3	51.5	21.5	7.3	4.0	32.8
	**2011**	9,792	3.4 ± 1.5	2.9 ± 1.2	1.2	32.3	48.6	12.3	3.5	2.1	66.5	3.3 ± 1.3	0.6	19.5	47.3	22.6	5.6	4.3	32.6
	**2012**	9,747	3.3 ± 1.3	2.9 ± 1.0	1.0	34.5	47.7	12.0	3.0	1.8	64.5	3.3 ± 1.4	0.5	19.0	52.3	18.6	4.6	4.9	28.1
	**2013**	9,289	3.3 ± 1.5	2.9 ± 1.1	1.1	35.2	47.4	11.7	30	1.8	63.7	3.2 ± 1.2	0.8	25.1	47.6	15.8	6.5	4.2	26.6
	**2014**	9,095	3.2 ± 1.5	2.8 ± 1.1	1.3	42.3	42.8	10.2	2.9	1.6	57.4	3.2 ± 1.1	0.5	23.8	46.5	20.5	5.2	3.5	29.2
	**2015**	8,659	3.2 ± 1.4	2.8 ± 1.0	1.6	40.9	43.0	10.3	2.8	1.7	57.5	3.2 ± 1.2	1.0	23.1	49.7	17.7	4.7	3.8	26.2
**Hospital** (Missing: 193)	**A**	19,059	3.1 ± 1.7	2.5 ± 1.1	3.1	58.9	32.0	3.7	1.1	1.1	38.0	3.1 ± 1.3	1.3	27.5	51.7	12.3	3.1	4.0	19.5
	**B**	18,380	3.9 ± 1.3	3.5 ± 1.0	0.4	2.6	54.6	32.1	7.3	3.1	97.0	3.9 ± 1.0	0.0	0.6	35.5	45.2	12.9	5.8	63.9
	**C**	8,840	3.1 ± 1.0	2.8 ± 0.8	0.3	28.8	64.7	4.1	1.1	1.0	70.9	3.2 ± 0.8	0.0	0.7	75.4	10.3	3.5	2.2	16.0
	**D**	3,330	4.0 ± 1.5	3.4 ± 1.1	0.5	7.1	61.1	23.9	4.4	3.1	92.5	3.8 ± 1.2	0.9	4.4	36.0	42.1	10.5	6.1	58.8
	**E**	6,673	3.5 ± 1.4	3.1 ± 1.0	0.8	20.4	63.9	9.0	2.7	3.3	78.3	3.7 ± 1.0	0.5	6.5	61.0	16.5	6.1	9.4	32.0
	**F**	5,723	3.2 ± 1.2	2.7 ± 0.8	0.8	38.6	53.9	4.9	1.4	0.7	60.6	3.0 ± 1.0	0.6	28.7	56.0	9.7	1.7	3.4	14.7
	**G**	9,146	2.8 ± 1.3	2.4 ± 0.9	1.5	66.0	25.6	4.0	1.7	1.2	32.5	2.8 ± 1.3	0.6	49.9	34.3	8.9	4.1	2.2	15.1
	**H**	11,681	3.0 ± 1.2	2.7 ± 0.9	1.1	39.4	47.4	9.4	1.7	0.9	59.4	3.2 ± 1.0	0.7	18.3	55.5	18.5	4.5	2.5	25.5
	**I**	6,047	3.6 ± 1.4	3.1 ± 1.0	1.5	16.5	63.0	12.9	3.6	2.5	82.0	3.5 ± 1.2	0.8	5.5	57.5	23.0	7.7	5.5	36.2
	**J**	12,035	3.3 ± 1.7	2.9 ± 1.3	1.2	43.0	36.5	11.8	4.1	3.3	55.8	3.4 ± 1.5	0.7	25.4	38.0	19.9	8.9	7.2	36.0
	**K**	8,027	3.5 ± 1.2	3.1 ± 0.9	0.8	17.7	63.8	12.4	3.8	1.7	81.5	3.4 ± 1.0	0.3	7.1	58.7	23.2	6.5	4.2	33.9
	**L**	12	4.9 ± 4.9	NA	NA	NA	NA	NA	NA	NA	NA	NA	NA	NA	NA	NA	NA	NA	NA
**TOTAL**		**109,246**	**2.9** ± 1.1	**2.9** ± 1.1	**1.3**	**34.9**	**48.0**	**11.5**	**2.5**	**1.9**	**64.4**	**3.3** ± 1.1	**0.6**	**18.8**	**48.6**	**21.0**	**6.4**	**4.6**	**32.0**

#### Maternal health factors

Table 2 displays the classes of clinical explanatory factors related with the maternal health domain: mother’s age, hypertension/diabetes, amniocentesis, villi sample, fetoscopy, pre-delivery LoS, presentation, labour induction, neonatal status, number of obstetric checks performed, number of ultrasound (US) scans performed. In CEDAP fetal presentation is classified as follows: vertex; breech; shoulder; face; brow; Bregma; other. The group “other” was excluded from the analysis and coded as missing. A category named “cefalic” was created to incorporate vertex, face, brow and Bregma. Since shoulder presentation is incompatible with SVD and births by IVD are exceptional procedures requiring advanced obstetric craft, 39 shoulder presentations delivering by SVD and 1 shoulder presentation delivering by IVD were reclassified as cefalic. Leaving aside possible misclassification issues (which should not be ruled out), a transverse lie of the fetus will in fact ultimately result in a vaginal birth, either by spontaneous or manual version of the fetus to longitudinal lie.

**Table 2:** Distibution of length of stay (LoS) after childbirth by maternal health factors. Number; mean LoS (M) ± standard deviation (SD); row percentage (row %). NA = Not applicable.

FACTORS		STRATA	ALL BIRTHS		VAGINAL DELIVERY MODE	
			Number	M ± SD (days)	SPONTANEOUS (N = 75,497)	INSTRUMENTAL (N = 7,281)
					LoS >2 days (Row %)	LoS > 3 days (Row %)
**Delivery mode** (Missing: 1)		Spontaneous	75,497	2.9 ± 1.1	64.4	
		Instrumental	7,281	3.3 ± 1.3		32.0
		Caesarean	26,467	4.7 ± 1.7		
**Mother Age** (years) (Missing: 32)		15–19	1,254	3.4 ± 1.5	72.0	31.5
		20–24	9,485	3.3 ± 1.5	68.7	34.7
		25–29	23,675	3.3 ± 1.4	65.6	27.8
		30–34	38,381	3.3 ± 1.4	63.5	32.5
		35–39	28,860	3.4 ± 1.5	62.8	32.6
		40–44	7,214	3.6 ± 1.6	62.8	37.9
		45+	345	4.3 ± 2.3	74.8	40.0
**Hypertension/diabetes** (Missing: 63)		No	106,690	3.3 ± 1.4	64.2	31.8
		Yes	2,493	4.5 ± 2.4	74.6	41.8
**Villi sample** **(**Missing: 6)		No	105,993	3.3 ± 1.5	64.3	32.0
		Yes	4,247	3.5 ± 1.5	67.5	32.0
**Amniocentesis** (Missing: 6)		No	91,986	3.3 ± 1.5	64.2	31.2
		Yes	17,254	3.5 ±1.5	65.5	36.6
**Fetoscopy**(Missing: 6)		No	108,892	3.3 ± 1.5	64.4	32.0
		Yes	348	3.4 ±1.5	66.8	37.9
**Number of** **obstetric checks** (Missing: 1)		<4	20,856	3.5 ± 1.6	65.4	35.3
		4–7	65,800	3.3 ± 1.4	66.6	32.3
		8+	22,589	3.3 ± 1.5	56.7	28.8
**Number of US scans** **in pregnancy** (Missing: 7)		<4	19,003	3.1 ± 1.4	56.5	25.7
		4–5	52,873	3.3 ± 1.4	62.6	28.6
		6+	37,363	3.6 ± 1.6	72.1	36.9
**Analgesia** (Missing: 184)		No	89,536	3.3 ± 1.5	63.6	28.5
		Yes	19,526	3.3 ± 1.4	67.7	38.1
**Labour induction** (Missing: 68)		No	81,859	2.9 ± 1.1	64.1	31.0
		Yes	27,319	4.6 ± 1.7	82.6	51.5
**Neonatal status**		Liveborn	108,944	3.4 ± 1.5	64.5	32.1
		Stillborn	302	2.8 ± 2.8	12.3	6.7
**Pre-delivery LoS** (days) (Missing: 594)		<3	103,769	3.3 ± 1.4	64.3	31.8
		3–5	3,142	4.1 ± 2.0	68.8	35.6
		6+	1,741	5.0 ± 2.9	69.3	45.8
**Presentation** (Missing:181)	**Cefalic**	Spontaneous	75,118	2.9 ± 1.0	64.4	
		Instrumental	7,248	3.3 ± 1.3		32.0
	**Breech**	Spontaneous	368	3.0 ± 1.4	61.0	
		Instrumental	27	3.8 ± 1.6		48.2
	**Shoulder**	Spontaneous	0	NA	NA	NA
		Instrumental	0	NA	NA	NA

#### Child’s clinical factors fragility

We created a variable called “*Child’s size*” using anthropometric charts combining the distribution of four available factors: sex of child; parity, birth-weight and gestational age [28,29]. The variable was categorized in three levels: small for gestational age (SGA); appropriate for gestational age (AGA); large for gestational age (LGA).

Table 3 displays the classes of clinical factors of the child, in particular:

- Child’s size factors: gestational age; birthweight; placenta weight; child’s size; and
- Child’s fragility factors: Apgar score at 1 minute; Apgar score at 5 minutes; resuscitation; intensive care unit admission (ICU); multiple birth.

**Table 3:** Distibution of length of stay (LoS) after childbirth by clinical factors of the child. Number; mean LoS (M) ± standard deviation (SD); row percentage (row %).

FACTORS	STRATA		ALL BIRTHS		VAGINAL DELIVERY MODE	
			Number	M ± SD (days)	SPONTANEOUS (N = 75,497)	INSTRUMENTAL (N = 7,281)
					LoS > 2 days (Row %)	LoS > 3 days (Row %)
**CHILD’S SIZE FACTORS**						
**Gestational** **age** (weeks)	<29		563	4.7 ± 3.5	47.6	0
	29–32		1,130	4.6 ± 2.4	47.2	57.1
	33–36		6,217	4.6 ± 2.2	76.9	56.2
	37–40		82,637	3.3 ± 1.3	63.9	31.5
	41+		18,699	3.2 ± 1.3	64.2	31.1
**Birthweight** (gr) (Missing: 5)	<1000		525	4.8 ± 2.8	49.0	52.9
	1,000–1,499		668			
	1,500–1,999		1,330			
	2,000–2,499		4,524	4.6 ± 2.2	78.1	42.8
	2,500–3,999		95,954	3.3 ± 1.3	64.3	31.5
	4,000–4,499		6,576	3.3 ± 1.3	62.2	35.1
	4,500+		664			
**Placenta weight** (gr) (Missing: 172)	<500		22,862	3.5 ± 1.7	68.1	32.5
	500–599		35,744	3.2 ± 1.3	63.7	31.2
	600–999		49,048	3.3 ± 1.4	63.1	32.3
	1,000–1,500		1,420	4.9 ± 2.1	70.8	57.1
Child’s size *	SGA		9,122	3.7 ± 1.7	68.6	34.4
	AGA		88,138	3.3 ± 1.4	63.8	30.9
	LGA		11,986	3.4 ± 1.4	66.0	38.1
**CHILD’S FRAGILTY FACTORS**						
**Apgar score** **1 minute**	<7		6,807	4.0 ± 2.3	65.8	32.9
	7+		102,439	3.3 ± 1.4	64.3	31.9
**Apgar score** **5 minute**	<8		2,386	4.1 ± 2.6	56.7	38.3
	8+		106.860	3.3 ± 1.4	64.5	31.8
**ICU admission** (Missing: 221)	No		103,900	3.3 ± 1.4	64.4	32.1
	Yes		5,125	4.5 ± 2.5	62.8	31.5
**Resuscitation** (Missing: 54)	No		106,774	3.3 ± 1.4	64.4	32.0
	Yes		2,418	4.5 ± 2.7	63.5	33.1
**Multiple births** (Missing: 898)	Singleton	Female	51,806	3.3 ± 1.4	64.3	31.1
		Male	54,797			
	Twins or more		1,745	5.2 ± 2.0	74.0	58.3

* SGA = Small for Gestational Age; AGA = Appropriate for Gestational Age; LGA = Large for Gestational Age

#### Obstetric history

Table 4 displays the classes of factors pertaining with the obstetric history of the woman: previous livebirths; previous CS; pervious stillbirths; previous pre-term births; previous spontaneous abortions; previous neonatal deaths.

**Table 4:** Distribution of length of stay (LoS) after childbirth by socio-demographic and obstetric history factors. Number (N); mean LoS (M) ± standard deviation (SD); row percentage (row %).

FACTORS		STRATA		ALL BIRTHS		VAGINAL DELIVERY MODES	
				Number	M ± SD (days)	SPONTANEOUS (N = 75,497)	INSTRUMENTAL (N = 7,281)
						LoS > 2 days (Row %)	LoS > 3 days (Row %)
**SOCIO-DEMOGRAPHIC FACTORS**							
**Father’s age** (years) (Missing: 1,949)		15–19		199	3.3 ± 1.3	74.7	36.4
		20–24		2,798	3.3 ± 1.4	67.9	34.3
		25–29		12,982	3.3 ± 1.4	66.2	28.1
		30–34		31,601	3.3 ± 1.4	65.5	31.6
		35–39		34,560	3.3 ± 1.5	63.3	33.6
		40–44		17,866	3.4 ± 1.5	62.3	31.3
		45–49		5,353	3.5 ± 1.5	63.1	32.2
		50–54		1,361	3.5 ± 1.7	61.6	37.5
		55+		577	3.5 ± 1.5	66.6	42.9
**Mother’** **snationality** (Missing: 116)		EU	Italian	86,083	3.3 ± 1.4	63.9	32.1
			Non-Italian	5,983	3.2 ± 1.2	63.9	27.6
		Non-EU		17,064	3.5 ± 1.7	67.1	33.2
**Marital status** (Missing: 8,155)		Not married		12,036	3.4 ± 1.6	62.1	28.9
		Married		70,340	3.3 ± 1.5	63.5	33.3
		Separated		1,136	3.5 ± 1.9	58.9	33.7
		Widow		82			
		Divorced		669			
		Living together		16,846	3.3 ± 1.4	67.2	30.5
**Mother’s education** (Missing: 24)		University or more		29,150	3.3 ± 1.4	64.5	35.4
		Secondary		52,988	3.3 ± 1.4	65.0	31.3
		Junior Secondary		25,107	3.5 ± 1.6	62.6	29.1
		Primary/none		1,977	3.6 ± 1.7	67.4	33.3
**Father’s education** (Missing: 6,772)		University or more		18,542	3.4 ± 1.5	64.1	36.3
		Secondary		51,356	3.3 ± 1.4	64.6	33.1
		Junior Secondary		30,767	3.3 ± 1.5	63.2	30.1
		Primary/none		1,809	3.5 ± 1.6	67.5	32.2
**Mother’s occupation** (Missing: 34,592)		Self-e/Enterpreneur		9,037	3.3 ± 1.4	64.4	33.6
		Manager		2,145	3.4 ± 1.3	69.0	45.7
		Employed-Clerk		31,002	3.3 ± 1.4	65.7	32.0
		Blue Collar		12,836	3.4 ± 1.4	66.7	32.2
		Other (employed)		19,634	3.3 ± 1.4	60.6	29.4
**Father’s** **occupation** (Missing: 10,867)		Self-e/Enterpreneur		22,100	3.3 ± 1.4	63.9	33.2
		Manager		3,338	3.5 ± 1.4	69.8	38.6
		Employed-Clerk		22,53	3.3 ± 1.5	63.4	34.8
		Blue Collar		32,812	3.4 ± 1.5	65.9	31.3
		Other (employed)		17,592	3.3 ± 1.5	60.2	30.4
**Consaguinity**		No		109,110	3.4 ± 1.5	64.4	32.1
		Yes		147	3.0 ± 1.3	48.2	22.2
**OBSTETRIC HISTORY FACTORS**							
**Previous** **livebirths**		0		58,217	3.6 ± 1.5	75.3	34.8
		1		39,805	3.1 ± 1.3	54.6	20.2
		2		8,644	3.1 ± 1.4	50.1	16.1
		3		1,820	2.6 ± 1.6	48.4	12.5
		4+		755	3.5 ± 1.5	46.9	0.0
**Previous** **stillbirths**		0		108,502	3.3 ± 1.5	64.4	32.1
		1+		744	3.8 ± 1.6	66.2	25.0
**Previous** **caesarean sections**		0		100,003	3.3 ± 1.4	64.6	32.6
		1		8,097	3.9 ± 1.5	57.0	21.3
		2+		1,146	4.3 ± 1.4	85.3	25/0
**Previous** **pre-term babies** (Missing: 1,144)		0		105,774	2.3 ± 1.5	64.2	32.0
		1		2,041	3.5 ± 1.7	63.3	28.2
		2+		287	3.7 ± 1.7	61.3	30.0
**Previous spontaneous** **abortions**		0		92,694	3.4 ± 1.5	65.7	32.8
		1		12,555	3.2 ± 1.5	56.3	25.8
		2		2,897	3.3 ± 1.5	57.4	30.6
		3+		1,099	3.5 ± 1.7	61.7	29.3
**Previous** **neonatal deaths**		0		108,923	3.3 ± 1.5	64.4	32.0
		1+		323	3.7 ± 1.7	61.8	37.5

#### Socio-demographic background

Table 4 displays classes of socio-demographic factors: father’s age; mother’s nationality; marital status of the woman; mother’s education; mother’s occupation; father’s education; father’s occupation; consanguinity.

### Statistical analysis

The mean and proportion of LoS longer than our proposed ED benchmarks were calculated for all available factors and for both modes of vaginal deliveries.

We ran two sets of logistic regression analyses to identify factors associated with LoS longer than the ED cutoffs, defined as > 2 days for SVD and >3 days for IVD. We chose the most widely adopted ED benchmarks for SVD. We proposed a 3 days ED threshold for IVD, as a measure of compromise between SVD (2 days) and CS (4 days) cutoffs recommended by the American Academy of Pediatrics and the American College of Obstetricians and Gynecologists [18].

Hospital A was chosen as reference since it is a national excellence centre for maternal and child health. Moreover hospital A managed a higher volume of births (N = 19,059) in FVG during 2005–2015, had the second shortest mean LoS for SVD and the third shortest mean LoS for IVD.

Initially a logistic regression was run for each factor in turn, using LoS as endpoint and adjusting only for hospital. Significant factors and potential confounders were then selected to be included in the final multiple logistic regression model.

Some factors were deliberately dropped from the final multivariate logistic model for the following different reasons:

- Apgar score at 1 minute and resuscitation due to collinearity with Apgar score at 5 minutes and intensive care unit (ICU) admission respectively, which both had stronger effect size and we thought they were more plausible to be retained in the final model;
- child’s size, due to collinearity with birthweight and gestational age, both with stronger effect size;
- previous spontaneous abortions, as the relative effect was not consistent across the two vaginal delivery modes;
- father’s education, father’s occupation, marital status and pre-term history, since in addition of being affected by a large number of missing values, their significance was inconsistent across the two vaginal delivery modes and their effect size was negligible.

Results were expressed as odds ratios (OR) with 95% confidence interval (95%CI), comparing each stratum specific to the baseline reference category. Since the percentage of missing values was less than 10% for each factor analyzed, complete case analysis was adopted. Although the two multiple logistics models were adjusted for all factors, only the estimates for hospitals and calendar year are displayed in this paper.

Sensitivity analysis was then fitted by excluding marital status and pre-term history from both final multivariable logistic regression models.

Population attributable risks (PAR) is an epidemiologic measure widely used to evaluate the impact of modifiable factors on study populations, assuming there is a perfect intervention to remove such factors [30]. PAR (percentage variation) with 95%CI were therefore calculated for each maternity centre in the ideal scenario of having the same performance as hospital A during calendar year 2015 for both delivery modes. In addition to the latter criteria, PAR was also calculated assuming each hospital had the same performance of A during 2015 and considering only low risk pregnancies (low risk conditions for the mother as well as the newborn with potential impact on LoS), which we defined as pregnancies undergoing SVD and meeting simultaneously all the following criteria:

- Maternal age < 35 years;
- Mother without hypertension/diabetes;
- Singleton birth;
- Gestational age: 37–40 weeks;
- Birthweight: 2,500–3,999 g;
- Pre delivery LoS ≤2 days;
- No labour induction;
- Apgar score at 1 minute ≥7;
- Apgar score at 5 minute ≥8;
- Child not admitted to ICU;
- No resuscitation performed.

In addition to all the above criteria, our definition of low risk pregnancies in the calculation of PAR for IVD excluded also women administered with labor analgesia.

Consideration of the most recent estimates of deliveries (calendar year 2015) could allow to envisage future patterns of LoS.

Stata 14.2 (College Station, Texas, USA) was employed for the analysis.

## Results

A total of 109,810 birth records were available in our database. After excluding 260 duplicates, 293 births outside hospital and 11 births in minor centers without maternity services, we were left with a final number of 109,246 hospital birth records for the analysis (Fig 1). The total number of SVD in FVG during 2005–2015 was 75,497 (69.1%), total IVD were 7,281 (6.7%) and CS were 26,467 (24.2%). As mentioned earlier, CSs will not be be treated in this study.

In addition to the relevant number of births, Tables 1–4 display the distribution of LoS by explanatory factors in terms of mean and proportion of stays longer than the ED benchmarks for both vaginal delivery modes (spontaneous as well as instrumental).

Table 1 shows the mean LoS and the proportion of LoS > ED according to calendar year (upper panel) and facility centre (lower panel). The latter variations were considerably larger than the former ones for both SVD as well as IVD. The mean LoS of all hospitals consistently exceeded our proposed ED benchmarks for both delivery modes, with the only exception being hospital G for IVD.

Table 2 shows mild variation of mean LoS and proportion of LoS > ED among the strata of each explanatory factor, in particular maternal age, hypertension/diabetes, pre-delivery LoS, presentation, neonatal status.

Table 3 shows that the mean LoS was higher for gestational age less than 36 weeks, for birthweight less than 2,000 gr and for placentas weighing more than 1 Kg. Big placenta weights may mask the influence of multiple birth, which also showed a high mean LoS and high proportion LoS longer than ED.

Table 4 shows minor changes in mean LoS and probability of surpassing the ED cutoffs for socio-demographic factors of parents and major differences for obstetric history factors, in particular with increasing parity and with history of CS.

Table 5 shows factors associated with LoS longer than our proposed ED benchmarks, based on multivariable logistic regression analysis. Only estimates for hospital and calendar year (both adjusted for all other factors) are displayed, The adjusted OR of having a LoS higher than 2 days for SVD ranged from 2.39 (95%CI: 2.24–2.55) for hospital J up to 89.38 (95%CI: 78.49–101.78) for hospital B. Although with attenuated effect size, similar patterns were also observed for IVD. Moreover, a significant decreasing trend over time of LoS>ED was observed in the whole FVG for both delivery modes.

**Table 5:** Multiple logistic regression analysis. Outcome: length of hospital htay (LoS) longer than ED benchmarks (2 days for spontaneous vaginal deliveries; 3 days for instrumental vaginal deliveries). Effect estimates for hospital and calendar year adjusted for all other factors. Adjusted odds ratios (aOR*) and population attributable risks (PAR1^$^^,^ PAR2,** PAR3,^$^ PAR4**) with 95% confidence intervals (95%CI). NA = Not available; observations = complete (case analysis) observations.

FACTORS	STRATA	VAGINAL DELIVERY MODE					
		SPONTANEOUS			INSTRUMENTAL		
		aOR (95%CI) (LoS >2 vs. ≤ 2) (73,281 observations)	PAR1 (95%CI)	PAR2 (95%CI)	aOR (95%CI) (LoS >3 vs. ≤ 3) (7,050 observations)	PAR3 (95%CI)	PAR4 (95%CI)
**HOSPITAL**	**A**	reference	reference	reference	reference	reference	reference
	**B**	89.38 (78.49; 101.78)	+64.5% (+63.4%; +65.6%)	+65.8% (+64.6%; +67.0%)	7.90 (6.38; 9.78)	+44.8% (+41.0%; +48.5%)	+43.2% (+39.4%; +46.9%)
	**C**	4.86 (4.51; 5.23)	+37.5% (+35.9%; +39.0%)	+38.1% (+36.5%; +39.7%)	0.83 (0.59; 1.17)	-0.0% (-5.2%; +4.3)	-0.3% (-4.3%; +3.7.%)
	**D**	26.47 (22.35; 31.46)	+59.0 (+57.5%; +60.6%)	+60.2% (+58.6%; +61.8%)	7.85 (5.08; 12.12)	+44.7% (+35.1%; +53.4%)	+43.0 (+33.0%; +52.1%)
	**E**	8.40 (7.68; 9.19)	+46.7% (+45.1%; +48.2%)	+47.5 (+45.9%; +49.1%)	2.21 (1.67; 2.94)	+16.1% (+10.7%; +21.5%)	+14.5% (+9.5%; +19.4%)
	**F**	2.93 (2.69; 3.20)	+27.4% (+25.6%; +29.3%)	+27.9% (+26.0%; +29.8%)	0.79 (0.58; 1.08)	-1.1% (-5.2%; +3.1%)	-1.0 (-4.3%; +2.8%)
	**G**	0.77 (0.72; 0.83)	-1.0 (-2.2; +1.0%)	-1.0% (-2.1%; +1.0%)	0.72 (0.56; 0.95)	-2.2% (-5.7%; +1.4%)	-1.7 (-4.7%; +1.3%)
	**H**	2.78 (2.61; 2.96)	+26.3% (+24.8; +27.7%)	+26.7% (+25.2%; +28.2%)	1.53 (1.21; 1.94)	+9.0% (+5.0%; +13.0%)	+7.9% (+4.4%; +11.4%)
	**I**	10.42 (9.49; 11.44)	+49.7% (+48.2%; +51.2%)	+50.6% (+49.0%; +52.1%)	2.85 (2.15; 3.78)	+21.5% (+15.8%; +27.1%)	+19.6% (+14.2%; +24.9%)
	**J**	2.39 (2.24; 2.55)	+23.1% (+21.6%; +24.6%)	+23.5% (+22.0%; +25.0%)	2.56 (2.03; 3.23)	+19.2% (+14.8; +23.5%)	+17.3% (+13.4%; +21.3%)
	**K**	10.30 (9.45 11.21)	+49.5% (+48.1%; +51.0)	+50.4% (+48/9%; +51.9%)	2.41 (1.88; 3.10)	+17.9% (+13.2%; +22.5%)	+16.1% (+11.8%; +20.4%)
	**L**	NA	NA	NA	NA	NA	NA
**Calendar year (2005–2015)**		0.96 (0.95; 0.96)			0.97 (0.95; 0.99)		

* Multiple logistic regression model adjusted for: **Health care setting and time-frame factors** (hospital and calendar year); **Maternal health factors** (mother’s age; hypertension/diabetes; amniocentesis; number of obstetric checks; number of ultrasound scans performed; no labour induction; labour analgesia; neonatal status; presentation; pre-delivery LoS); **Child’s fragility factors** (Apgar score at 5 minutes; ICU admission; multiple birth); **Child’s size factors** (gestational age; birthweight; placenta weight); **Obstetric history factors** (parity; history of caesarean sections); **Socio-demographic factors** (father’s age; mother’s nationality; mother’s educational level)

^$^ **Population Attributable Risk 1 (PAR 1) and 3 (PAR 3).** Proportional variation of LoS < ED after childbirth in the ideal scenario each hospital would be performing as hospital A during calendar year 2015

** **Population Attributable Risk 2 (PAR 2) and 4 (PAR4).** Proportional variation of LoS < ED after childbirth in the ideal scenario each hospital would be performing as hospital A during calendar year 2015. Estimations of PAR2 and PAR4 calculated only for low risk pregnancies, defined as conditions of the mother and/or the newborn simultaneously meeting all the following criteria: for spontaneous vaginal deliveries (PAR 2): mother’s age<35; no resuscitation performed; child not admitted to ICU; singleton birth; Apgar score at 1 minute ≥7; Apgar score at 5 minutes ≥8; no labour induction; no women affected by hypertension/diabetes; birthweight: 2,500–3,999gr; gestational age: 37–40 weeks; pre delivery LoS ≤2 days; for instrumental vaginal deliveries (PAR 4): in addition to all above criteria, the calculation of PAR4 was restricted to women not administered with labour analgesia.

As can be seen also from Table 5, in the ideal scenario each hospital would be performing as hospital A during calendar year 2015, a significant increase in ED rate for SVD would be seen for all hospitals but G (which conversely would have a slightly smaller, non-significant ED rate). The proportional increase in LoS<ED for SVD would range from 23.1% (centre J) up to 64.5% (centre B), and would be +59.0%, +49.7%, +49.5%, +46.7%, +37.5%, +27.4% and +26.3% for centres D, I, K, E, C, F and H respectively (PAR1). Almost overlapping figures of PAR for SVD were obtained by restricting the analysis to low risk pregnancies (PAR2). For IVD, the pattern of PAR (PAR3 and PAR4) was rather similar to SVDs’, although slightly mitigated (Table 5).

S1 Table shows the results of the sensitivity analysis. The removal of pre-term history and marital status from the final logistic regression model had little impact on the effect size of all other factors.

## Discussion

### Key findings

This population-based study analyzed all hospital births in FVG from 2005 to 2015. During these 11 years, the number of births in the region significantly diminished over time and the pooled mean LoS was 2.9 days for SVD and 3.3 days for IVD.

In FVG we found a decreasing trend over time in the proportion of women staying longer than the respective ED benchmarks for SVD as well as IVD. Nonetheless, the average regional LoS was still consistently higher than the ED benchmarks for both delivery modes. After removing the effect of all other factors, all regional hospitals but G were by far more likely than the referral hospital A to overcome the ED benchmarks for SVD. A similar, mitigated pattern was observed also for IVD.

In the ideal scenario of performing as hospital A during calendar year 2015, all FVG hospitals but G would have a significant increase in ED rate for both delivery modes, reaching the percentage of 64.5% and 59.0% for SVD in centres B and D, and being about 50% for facilities E, I and K (PAR1). Similar attenuated patterns were observed also for IVD (PAR 3). Almost the same figures were found in low risk pregnancies for SVD as well as IVD (PAR 2 and PAR 4). Hospital A managed the highest volume of SVD in FVG during 2005–2015 (17.8% = 13,445/75,383). However, despite having the second shortest mean LoS for SVD (2.5 days), 38.0% (= 5,090/13,409) women still remained admitted more than 2 days following childbirth (data not shown in any tables). The latter proportion reduced to 34.6% (= 1,925/5,568) among the subgroup of low risk pregnancies, and was still 34.0% (= 2,710/ 7,982) by including also women in the age band 35–39 years (relaxed definition of low risk pregnancies). Therefore, there seems to be room for substantial performance improvement in FVG hospitals, particularly for SVD.

### Generalizability

The pooled mean LoS for SVD was 2.9 days in FVG, shorter than the average figures most recently reported for the whole of Italy (3.4 days) [4,19]. However, Organization for Economic Cooperation and Development (OECD) data from other countries with Beveridgean health systems similar to Italy’s reported average LoS for SVD shorter than FVG, in particular Sweden (2.3 days), Ireland (2.0 days), the Netherlands (1.9 days) and the UK (1.5 days) [19].

Since our results were controlled for the effect of all other factors, the between-hospital variability we found is likely attributable to the single health care provider itself. The Diagnosis Related Group (DRG) system, introduced in Italy in 1995, was designed to improve hospital efficiency and contain average LoS, a goal generally attained in several high income countries, including Italy [31]. However, despite the prospective payment health system based upon capitation grants in place in Italy, it can be argued that some maternity centres of FVG may still have had convenience in extended hospitalizations after childbirth, to show higher daily rates of bed occupancy as a measure of health care activity to negotiate their allocated budgets with the regional government. Although, fear of medico-legal backlashes, internal organizational malfunctions of hospital and scarce attention of ward staff on performance efficiency shall not be ruled out [31–34].

Variation in ED rates by region and matenity centre also exists elsewhere. For instance, in England it is estimated that currently almost 30% women remain hospitalized more than 2 days following childbirth [35]. In a recent Danish study on 2,786 pregnant women, the proportion discharged within 48h was almost 60%, with 25% of them being discharged between 13h and 50h after childbirth, and 34% remaining admitted for less than 12h (very early discharge, VED). Seventy percent of the latter Danish women expected ED, hinting that this approach has become established in Denmark [8]. ED and VED are the norm in Scandinavia [15] and are also common in many other high-income countries such as Australia, Canada, UK and Ireland [16,36]. In a recent Italian study, a significant increase in ED for SVD was found. However, the definition of ED threshold adopted was 3 days, hence longer than ours [17]. The latter Italian study reported higher odds of ED with increasing number of hospital births on the birthday of the index child, suggesting that ED may become feasible if higher hospital efficiency is needed in terms of bed turnover [17]. A higher number of birth per day generally determines shorter LoS, but seems not to translate into increased readmission rates [37].

### Prospects

Italian women are still referred almost exclusively to hospitals both for pre-natal and post-natal care. Much of this care could instead be managed by health districts through general practitioners and community midwives, who could also provide home visits, especially during postnatal care [38]. This could also pave the way for optional home births, a practice rather popular in the Netherlands but still culturally unaccepted in Italy [39,40]. Effective postnatal care following ED would need adequate investment in community services to ensure continuity of care [36,41,42]. Northern European countries have invested more in midwifery care [36,38,40]. For instance, Scandinavian countries employ models of care based upon birth centres [15,40]. These centres (designed to simulate a domestic environment) are managed by midwives, welcome parental involvement, encourage natural childbirth and minimize postnatal facility stay. Midwives provide antenatal, perinatal, and postnatal home care. Standard care involving obstetricians is offered only in case of complications or if the woman desires analgesia [40]. In the UK prenatal care until childbirth is managed by midwives and women have the option to deliver in hospital or in midwifery led units, where the new mothers are discharged usually within 6 to 48 hours following delivery, receiving four to five postnatal midwifery home visits afterwards [36]. In some areas of Italy, experimental programs are being conducted where ED is accompanied by follow-up phone calls to monitor the postnatal conditions of the woman and early pediatric care is provided [17].

The FVG Regional Health System (RHS) has recently been revised to increase quality and efficiency of the health care delivered. A higher level of coordination among health services was introduced with the aim of overcoming fragmentation and meaningless competition between health-care facilities. Maternity services are now required to ensure at least 1,000 births per year in order to maintain their status and all complex cases should be forwarded to referral centres. The revised RHS has allocated substantial resources to set up intermediate health services (country hospitals, community rehabilitation centres and care homes) for cost-effective management of long-term conditions following “protected” discharge from hospitals [43]. Nonetheless, this integrated system of secondary and primary care services was not extended to maternal and child health. Considering child delivery is also one of the major causes of hospitalization in high-income countries, an organizational revision of the RHS is recommended [17].

### Strengths and limitations

The database we analyzed is highly reliable, since it contains administrative hospital data collected by trained health care staff. Any long LoS is therefore very unlikely to be an outlier, but rather reflects the clinical condition of the woman requiring extended hospitalization. Nevertheless, the proportion of women remaining admitted for more than 10 days was just 0.4% (= 422/108,469) in our study. Since it comprises all births records of FVG, the design and methods of this study are strong. The size of the database and the large number of variables available allows this work to bring important and accurate conclusions. Furthermore, the percentage of missing values was negligible, and mainly pertained to socio-demographic factors. It can be argued that a proportion of women may have been reluctant to disclose some personal information. Completeness of clinical data was instead very close to 100%.

Although this study focused on a particular Italian region (FVG), we believe our findings can be to some extent generalized to other Italian and European regions with health systems similar to FVG. We employed an ED threshold for IVD which has not been validated yet, but we believe it makes sense, since it is a compromise between the internationally recognized ED cutoffs for SVD and CS. Moreover, the methodology we proposed to contrast hospital performance is sound as it took into account a considerable number of determinants, including clinical factors, which could also be applied in the evaluation of different types of health systems. The innovative conceptual framework we devised to explain the relationship between various determinants and LoS is a significant advance from previous models [26].

## Conclusions

Despite a decreasing trend in hospitalization length for both vaginal delivery modes over time, women probably still stayed longer than needed in FVG hospitals. In low risk pregnancies, ED followed by community midwifery care may be a realistic and acceptable alternative model than standard delivery. Offering women the option of ED (in line with other European countries with healthcare systems similar to Italy’s) could not only meet the respectful preference of a proportion of Italian women, but could also reduce the risk of nosocomial infections, increase patient satisfaction with the health care services, prevent stress and sleeping disorders in the woman, contribute to improved performance of Italian hospitals and, ultimately, also contain healthcare costs associated with childbirth [44]. ED followed by domiciliary care is more cost-effective than standard delivery [38,45,46]. Furthermore, although the impact of ED on maternal and newborn outcomes is still inconclusive [33,35,47–50], there is evidence that ED is safe for full-term as well as early term babies [37].

The systematic methodology we proposed for FVG could be applied for confirmatory studies in other geographical areas, possibly at country level. In the future it would be useful to correlate our findings (and eventual applications of ED policies) with cost effective analysis and maternal/child health outcomes, employing also patient satisfaction surveys.

## Supporting information

S1 Fig Certificate of delivery care (CEDAP). (DOC)

S1 Table Sensitivity analysis. Sensitivity model A: multiple logistic regression analysis adjusted for hospital and all other factors Including pre-term history and marital status. Sensitivity model B: multiple logistic regression analysis adjusted for hospital and all other factors, but pre-term history and marital status. Odds ratio (OR) with 95% confidence interval (95%CI); LoS = length of hospital stay; NA = not available; observations = complete (case analysis) observations.
(DOCX)
