# Correction: Length of stay following vaginal deliveries: A population based study in the Friuli Venezia Giulia region (North-Eastern Italy), 2005-2015

**DOI:** 10.1371/journal.pone.0213664

**Published:** 2019-03-06

**Authors:** Luca Cegolon, Oona Campbell, Salvatore Alberico, Marcella Montico, Giuseppe Mastrangelo, Lorenzo Monasta, Luca Ronfani, Fabio Barbone

There is an error in the caption for Fig 1. Please see the complete, correct Fig 1 caption here.

**Fig 1:**
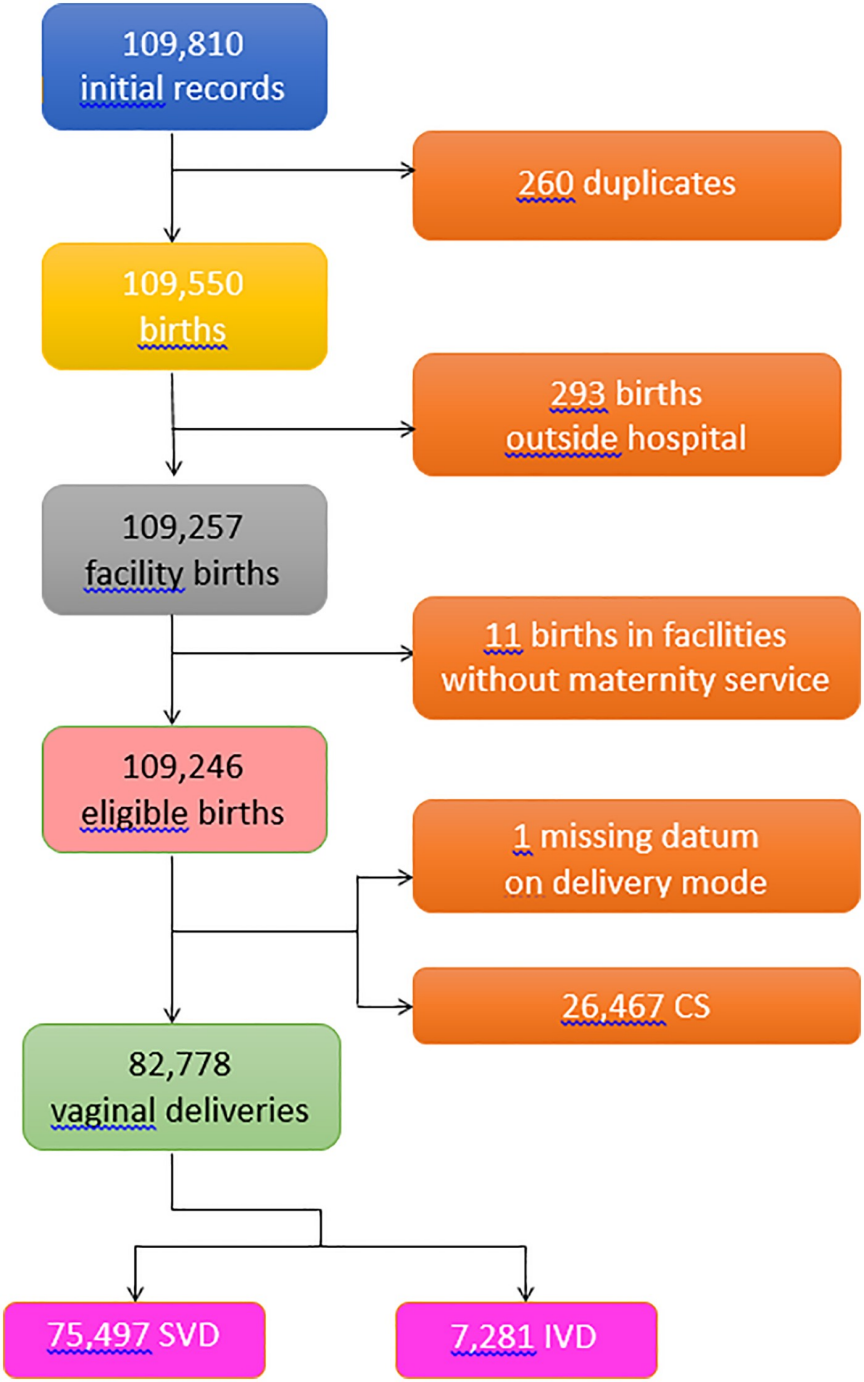
Flowchart displaying the various criteria applied to the initial database to obtain the final number of hospital records available for the analysis. SVD = spontaneous vaginal deliveries; IVD = instrumental vaginal deliveries; CS = cesarean sections.

There is an error in the caption for Fig 2. Please see the complete, correct Fig 2 caption here.

**Fig 2:**
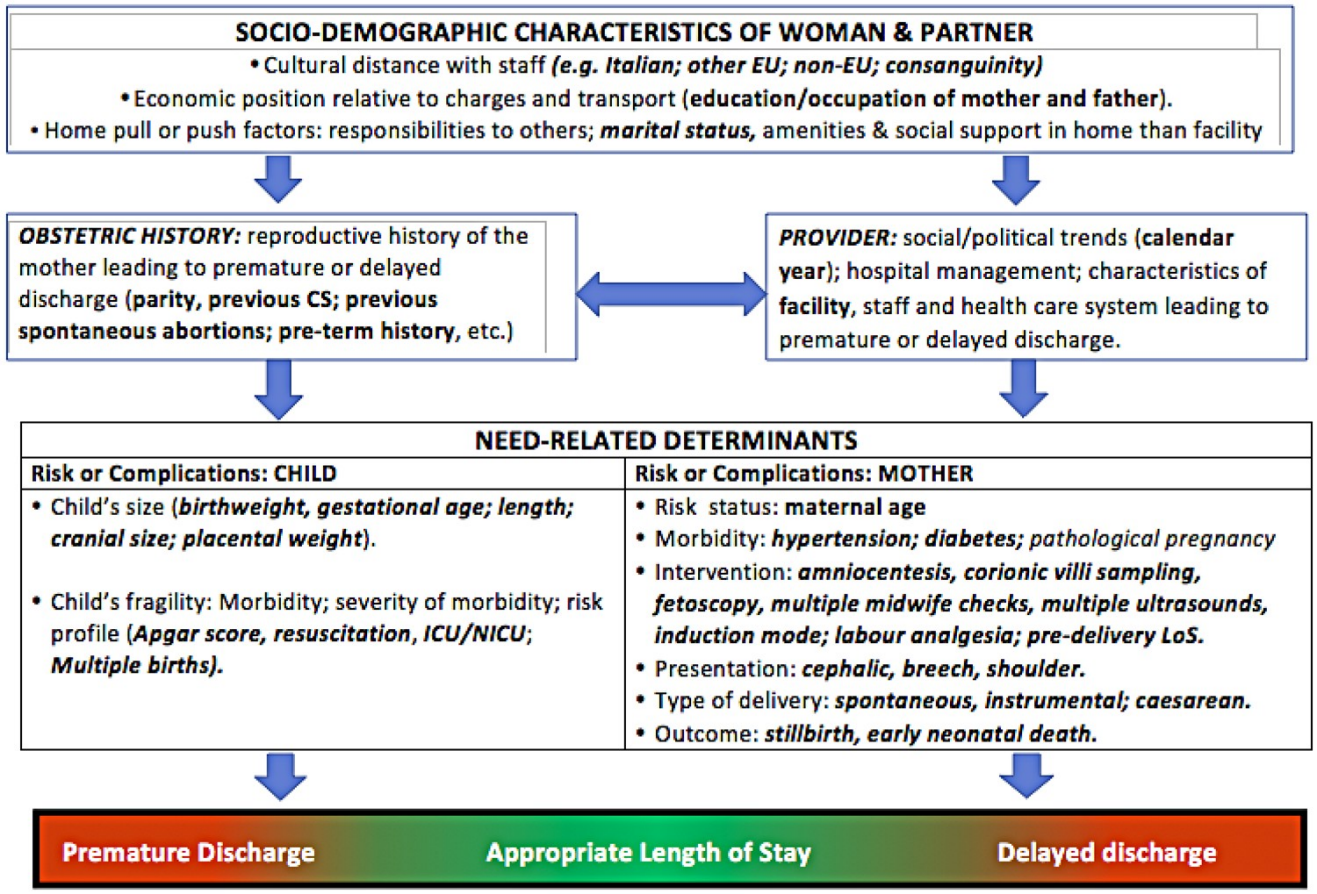
Conceptual framework explaining the relationship between various determinants and length of hospital stay after childbirth (LoS).

There is an error in Table 2. Please see the complete, correct Table 2 here.

**Table 2:** Distribution of length of stay (LoS) after childbirth by maternal health factors. Number; mean LoS (M) ± standard deviation (SD); row percentage (row %). NA = Not applicable.

FACTORS		STRATA	ALL BIRTHS		VAGINAL DELIVERY MODE	
			Number	M ± SD (days)	SPONTANEOUS (N = 75,497)	INSTRUMENTAL (N = 7,281)
					LoS >2 days (Row %)	LoS > 3 days (Row %)
**Delivery mode** (Missing: 1)		Spontaneous	75,497	2.9 ± 1.1	64.4	
		Instrumental	7,281	3.3 ± 1.3		32.0
		Caesarean	26,467	4.7 ± 1.7		
**Mother Age** (years) (Missing: 32)		15–19	1,254	3.4 ± 1.5	72.0	31.5
		20–24	9,485	3.3 ± 1.5	68.7	34.7
		25–29	23,675	3.3 ± 1.4	65.6	27.8
		30–34	38,381	3.3 ± 1.4	63.5	32.5
		35–39	28,860	3.4 ± 1.5	62.8	32.6
		40–44	7,214	3.6 ± 1.6	62.8	37.9
		45+	345	4.3 ± 2.3	74.8	40.0
**Hypertension/diabetes** (Missing: 63)		No	106,690	3.3 ± 1.4	64.2	31.8
		Yes	2,493	4.5 ± 2.4	74.6	41.8
**Villi sample** (Missing: 6)		No	105,993	3.3 ± 1.5	64.3	32.0
		Yes	4,247	3.5 ± 1.5	67.5	32.0
**Amniocentesis** (Missing: 6)		No	91,986	3.3 ± 1.5	64.2	31.2
		Yes	17,254	3.5 ±1.5	65.5	36.6
**Fetoscopy** (Missing: 6)		No	108,892	3.3 ± 1.5	64.4	32.0
		Yes	348	3.4 ±1.5	66.8	37.9
**Number of obstetric checks** (Missing: 1)		<4	20,856	3.5 ± 1.6	65.4	35.3
		4–7	65,800	3.3 ± 1.4	66.6	32.3
		8+	22,589	3.3 ± 1.5	56.7	28.8
**Number of US scans in pregnancy** (Missing: 7)		<4	19,003	3.1 ± 1.4	56.5	25.7
		4–5	52,873	3.3 ± 1.4	62.6	28.6
		6+	37,363	3.6 ± 1.6	72.1	36.9
**Labour analgesia** (Missing: 184)		No	89,536	3.3 ± 1.5	63.6	28.5
		Yes	19,526	3.3 ± 1.4	67.7	38.1
**Labour induction** (Missing: 68)		No	81,859	2.9 ± 1.1	64.1	31.0
		Yes	27,319	4.6 ± 1.7	82.6	51.5
**Neonatal status**		Liveborn	108,944	3.4 ± 1.5	64.5	32.1
		Stillborn	302	2.8 ± 2.8	12.3	6.7
**Pre-delivery LoS** (days) (Missing: 594)		<3	103,769	3.3 ± 1.4	64.3	31.8
		3–5	3,142	4.1 ± 2.0	68.8	35.6
		6+	1,741	5.0 ± 2.9	69.3	45.8
**Presentation** (Missing:181)	**Cefalic**	Spontaneous	75,118	2.9 ± 1.0	64.4	
		Instrumental	7,248	3.3 ± 1.3		32.0
	**Breech**	Spontaneous	368	3.0 ± 1.4	61.0	
		Instrumental	27	3.8 ± 1.6		48.2
	**Shoulder**	Spontaneous	0	NA	NA	NA
		Instrumental	0	NA	NA	NA

There are errors in Table 5. Please see the correct Table 5 here.

**Table 5:** Multiple logistic regression analysis. Outcome: length of hospital stay (LoS) longer than ED benchmarks (2 days for spontaneous vaginal deliveries; 3 days for instrumental vaginal deliveries). Effect estimates for hospital and calendar year adjusted for all other factors. Adjusted odds ratios (aOR*) and population attributable risks (PAR1^$^, PAR2,** PAR3,^$^ PAR4**) with 95% confidence intervals (95%CI). NA = Not available; observations = complete (case analysis) observations.

FACTORS	STRATA	VAGINAL DELIVERY MODE					
		SPONTANEOUS			INSTRUMENTAL		
		aOR (95%CI) (LoS >2 vs. ≤ 2) (73,281 observations)	PAR1 (95%CI)	PAR2 (95%CI)	aOR (95%CI) (LoS >3 vs. ≤ 3) (7,050 observations)	PAR3 (95%CI)	PAR4 (95%CI)
**HOSPITAL**	**A**	reference	reference	reference	reference	reference	reference
	**B**	89.38 (78.49; 101.78)	+64.5% (+63.4%; +65.6%)	+65.8% (+64.6%; +67.0%)	7.90 (6.38; 9.78)	+44.8% (+41.0%; +48.5%)	+43.2% (+39.4%; +46.9%)
	**C**	4.86 (4.51; 5.23)	+37.5% (+35.9%; +39.0%)	+38.1% (+36.5%; +39.7%)	0.83 (0.59; 1.17)	-0.0% (-5.2%; +4.3)	-0.3% (-4.3%; +3.7.%)
	**D**	26.47 (22.35; 31.46)	+59.0 (+57.5%; +60.6%)	+60.2% (+58.6%; +61.8%)	7.85 (5.08; 12.12)	+44.7% (+35.1%; +53.4%)	+43.0 (+33.0%; +52.1%)
	**E**	8.40 (7.68; 9.19)	+46.7% (+45.1%; +48.2%)	+47.5 (+45.9%; +49.1%)	2.21 (1.67; 2.94)	+16.1% (+10.7%; +21.5%)	+14.5% (+9.5%; +19.4%)
	**F**	2.93 (2.69; 3.20)	+27.4% (+25.6%; +29.3%)	+27.9% (+26.0%; +29.8%)	0.79 (0.58; 1.08)	-1.1% (-5.2%; +3.1%)	-1.0 (-4.3%; +2.8%)
	**G**	0.77 (0.72; 0.83)	-1.0 (-2.2; +1.0%)	-1.0% (-2.1%; +1.0%)	0.72 (0.56; 0.95)	-2.2% (-5.7%; +1.4%)	-1.7 (-4.7%; +1.3%)
	**H**	2.78 (2.61; 2.96)	+26.3% (+24.8; +27.7%)	+26.7% (+25.2%; +28.2%)	1.53 (1.21; 1.94)	+9.0% (+5.0%; +13.0%)	+7.9% (+4.4%; +11.4%)
	**I**	10.42 (9.49; 11.44)	+49.7% (+48.2%; +51.2%)	+50.6% (+49.0%; +52.1%)	2.85 (2.15; 3.78)	+21.5% (+15.8%; +27.1%)	+19.6% (+14.2%; +24.9%)
	**J**	2.39 (2.24; 2.55)	+23.1% (+21.6%; +24.6%)	+23.5% (+22.0%; +25.0%)	2.56 (2.03; 3.23)	+19.2% (+14.8; +23.5%)	+17.3% (+13.4%; +21.3%)
	**K**	10.30 (9.45 11.21)	+49.5% (+48.1%; +51.0)	+50.4% (+48/9%; +51.9%)	2.41 (1.88; 3.10)	+17.9% (+13.2%; +22.5%)	+16.1% (+11.8%; +20.4%)

* Multiple logistic regression model adjusted for: **Health care setting and time-frame factors** (hospital and calendar year); **Maternal health factors** (mother’s age; hypertension/diabetes; amniocentesis; number of obstetric checks; number of ultrasound scans performed; labour induction; labour analgesia; neonatal status; presentation; pre-delivery LoS); **Child’s fragility factors** (Apgar score at 5 minutes; ICU admission; multiple birth); **Child’s size factors** (gestational age; birthweight; placenta weight); **Obstetric history factors** (parity; history of caesarean sections); **Socio-demographic factors** (father’s age; mother’s nationality; mother’s educational level)

^$^ **Population Attributable Risk 1 (PAR 1) and 3 (PAR 3)**. Proportional variation of LoS < ED after childbirth in the ideal scenario each hospital would be performing as hospital A during calendar year 2015

** **Population Attributable Risk 2 (PAR 2) and 4 (PAR4)**. Proportional variation of LoS < ED after childbirth in the ideal scenario each hospital would be performing as hospital A during calendar year 2015. Estimations of PAR2 and PAR4 calculated only for low risk pregnancies, defined as conditions of the mother and/or the newborn simultaneously meeting all the following criteria: for spontaneous vaginal deliveries (PAR 2): mother’s age<35; no resuscitation performed; child not admitted to ICU; singleton birth; Apgar score at 1 minute ≥7; Apgar score at 5 minutes ≥8; no labour induction; no women affected by hypertension/diabetes; birthweight: 2,500–3,999gr; gestational age: 37–40 weeks; pre delivery LoS <2 days; for instrumental vaginal deliveries (PAR 4): in addition to all above criteria, the calculation of PAR4 was restricted to women not administered with labour analgesia.

In the Results, there is an error in the second sentence of the penultimate paragraph. The correct sentence is: The proportional increase in LoS<ED for SVD would range from +23.1% (centre J) up to +64.5% (centre B), and would be +59.0%, +49.7%, +49.5%, +46.7%, +37.5%, +27.4% and +26.3% for centres D, I, K, E, C, F and H respectively (PAR1).

In the Generalizability subsection of the Discussion, there is an error in the first sentence of the first paragraph. The correct sentence is: The pooled mean LoS for SVD was 2.9 days in FVG during the whole study period (2005–2015), shorter than the average figures most recently reported for the whole of Italy (3.4 days).

There are errors in the Methods.

The title of the subsection “Child’s clinical factors fragility” is incorrect. The correct subsection title is: “Child’s clinical factors.”

In the Statistical analysis subsection, there is an error in the third item of the first list. The correct third item is: previous spontaneous abortions, as the relative effect size was not consistent across the two vaginal delivery modes.

In the Maternal health factors subsection, there is an error in the first sentence of the first paragraph. The correct sentence is: Table 2 displays the classes of clinical explanatory factors related with the maternal health domain: mother’s age, hypertension/diabetes, amniocentesis, villi sample, fetoscopy, pre-delivery LoS, presentation, labour induction, labour analgesia, neonatal status, number of obstetric checks performed, number of ultrasound (US) scans performed.
