# Supplementary material for: Length of stay following vaginal deliveries: A population based study in the Friuli Venezia Giulia region (North-Eastern Italy), 2005-2015
Source: PLoS One. 2019 Jan 3;14(1):e0204919. doi: 10.1371/journal.pone.0204919 (PMC6317786; doi:10.1371/journal.pone.0204919)
Supplement: S1 Fig — (DOC) [file pone.0204919.s001.doc]

**REGIONE AUTONOMA FRIULI VENEZIA GIULIA**

CERTIFICATO DI ASSISTENZA AL PARTO

*Timbro del Reparto*

Io sottoscritto/a C.R.A. **_(1)_**

in qualità di: 1 medico ostetrico/a 2 ostetrico/a 3 altro

***certifico quanto segue:***

A) INFORMAZIONI SOCIO-DEMOGRAFICHE SULLA MADRE:

cognome da nubile **_(2)_** nome

nata a **_(2)_** pv **_(2)_** data di nascita **_(2)_** C.R.A. **_(2)_**

residente a **_(2)_** pv cittadinanza

condizione professionale/non professionale (barrare una sola voce):

1 occupata 4 studente

2 disoccupata 5 casalinga

3 in cerca di prima occupazione 6 altra condizione

posizione nella professione (se **occupata** barrare una sola voce):

1 imprenditore o libero professionista 4 lav. dip.: impiegato

2 altro lavoratore autonomo 5 lav. dip.: operaio

3 lav. dip.: dirigente/direttivo 6 altro lav. dip.

ramo di attività (se **occupata** barrare una sola voce):

1 agricoltura, caccia, pesca 4 pubblica amministrazione

2 industria 5 altri servizi privati

3 comm., pubbl. eserc., alberghi

titolo di studio conseguito (barrare una sola voce):

1 laurea 4 diploma di scuola media inferiore

2 diploma univ. o laurea breve 5 licenza elementare/nessun titolo

3 diploma di scuola media superiore

stato civile (barrare una sola voce):

1 non coniugata 4 vedova

2 coniugata 5 divorziata

3 separata 6 convivente

data di matrimonio (per coniugata/vedova):

cognome/nome coniuge nato il C.R.A. **_(1)_**

INFORMAZIONI SOCIO-DEMOGRAFICHE SUL PADRE **_(3)_**:

Mod. SAN. 11.KH. Ø1 - Ø1 - IMOCO s.p.a. - Villorba (TV) - Tel. 0422.9141

cognome nome

nato a pv data di nascita C.R.A. **_(1)_**

residente a pv cittadinanza

condizione professionale/non professionale (barrare una sola voce):

1 occupato 4 studente

2 disoccupato 5 casalingo

3 in cerca di prima occupazione 6 altra condizione

posizione nella professione (se **occupato** barrare una sola voce):

1 imprenditore o libero professionista 4 lav. dip.: impiegato

2 altro lavoratore autonomo 5 lav. dip.: operaio

3 lav. dip.: dirigente/direttivo 6 altro lav. dip.

ramo di attività (se **occupato** barrare una sola voce):

1 agricoltura, caccia, pesca 4 pubblica amministrazione

2 industria 5 altri servizi privati

3 comm., pubbl. eserc., alberghi

titolo di studio conseguito (barrare una sola voce):

1 laurea 4 diploma di scuola media inferiore

2 diploma univ. o laurea breve 5 licenza elementare/nessun titolo

3 diploma di scuola media superiore

INFORMAZIONI RELATIVE AD EVENTUALI GRAVIDANZE PRECEDENTI:

numero parti precedenti: numero delle I.V.G.:

numero totale dei nati vivi **_(4)_**: figli deceduti nel primo mese di vita **_(8)_**:

numero dei nati morti **_(5)_:** numero dei cesarei precedenti:

numero dei nati vivi pret. (<37s.) **_(6)_**: anno ultimo parto:

numero degli aborti spontanei **_(7)_**:

B) INFORMAZIONI RELATIVE ALL’ATTUALE GRAVIDANZA:

numero di visite di controllo in gravidanza (n.): ______

prima visita di controllo in gravidanza (sett.):

numero di ecografie in gravidanza:

indagini prenatali (barrare tutte le voci):

amniocentesi 1a si 1b no

prelievo villi coriali 2a si 2b no

fetoscopia/funicolocentesi 3a si 3b no

ecografia dopo la 22.esima sett. 4a si 4b no

decorso della gravidanza 1 fisiologico 2 patologico

ricoveri in ostetricia durante la gravidanza 1 si 2 no

ipertensione arteriosa in gravidanza trattata con farmaci 1 si 2 no

difetto di accrescimento fetale **_(9)_** 1 si 2 no

concepimento con tecniche di procreazione medico-assistita (se **sì** barrare una sola voce) **_(10)_**:

1 solo trattamento farmacologico per induzione dell’ovulazione

2 IUI trasferimento di gameti maschili nella cavità uterina

3 GIFT trasferimento di gameti nelle tube di Falloppio

4 FIVET fecondazione in vitro e trasferimento degli embrioni in utero

5 ICSI fecondazione in vitro tramite iniezione di spermatozoi nell’ovocita e trasferimento degli embrioni in utero

6 altre tecniche

C1) INFORMAZIONI SUL PARTO:

data ora

luogo del parto **_(11)_**:

1 istituto pubblico/privato 3 altra struttura

2 abitazione 4 altro

codice istituto denominazione

comune via n°

personale sanitario presente al parto al momento della nascita (barrare una o più voci):

1 ostetrico/a 4 anestesista

2 ostetrico-ginecologo 5 altro personale sanitario o tecnico

3 pediatra/neonatologo

altre presenze in sala parto (barrare una o più voci):

1 padre 2 persona di famiglia 3 persona di fiducia

numero di figli nati dal parto: maschi: femmine:

sett. di amenorrea prima del parto:

modalità del travaglio (barrare una sola voce) **_(12)_**:

1 spontaneo 2 indotto con farmaci 3 pilotato 4 senza travaglio

modalità del parto (barrare una sola voce) **_(13)_**:

1 spontaneo (vaginale senza ventosa o forcipe) 4 forcipe

2 cesareo in elezione o per fallita induzione 5 ventosa

3 cesareo in travaglio o in urgenza 6 altre modalità di parto per via vaginale

secondamento: 1 spontaneo 2 manuale e/o strumentale

peso della placenta: (gr.)

analgesia praticata durante il travaglio: 1 si 2 no

profilassi RH: 1 si 2 no

consanguineità tra madre e padre (se **sì** barrare una sola voce):

1 parenti 4° gr. (figli di fratelli/sorelle)

2 parenti 5° gr. (coniuge sposato con figlio/a di un suo primo cugino)

3 parenti 6° gr. (secondi cugini)

C2) INFORMAZIONI SUL NEONATO **_(14)_**:

cognome nome sesso

data di nascita: ora:

presentazione al parto (barrare una sola voce):

1 vertice 5 fronte

2 podice 6 bregma

3 spalla 7 altra

4 faccia

necessità di rianimazione **_(15)_**: 1 si 2 no

se **sì** indicare il personale sanitario che ha avviato la rianimazione nei primi 5 minuti (barrare una sola voce):

1 ostetrico/a 5 anestesista-rianimatore

2 medico ostetrico 6 altro medico

3 pediatra 7 altro ruolo profess. sanit.

4 neonatologo/a 8 altra persona

punteggi APGAR rilevati al: primo minuto: quinto minuto:

vitalità: 1 nato vivo **_(4)_** 2 nato morto **(compilare la sezione D) _(5)_**

necessità di trasferimento in terapia intensiva neonatale: 1 si 2 no

peso: (gr.) lunghezza: (cm) circ. cranica: (mm)

tipo dei genitali esterni **_(16)_**: 1 maschile 2 femminile 3 incerto

presenza di malformazioni **_(17)_**: 1 assenti 2 presenti **(compilare la sezione E)**

data di compilazione ora

**NOTE PER LA COMPILAZIONE DEL CERTIFICATO DI ASSISTENZA AL PARTO**

1. Il CRA del personale sanitario che compila il certificato e del padre, deve essere considerato facoltativo ed utile alla sola registrazione informatizzata dei dati.

2. In caso di donna che vuole partorire in anonimato (figlio non riconosciuto o di filiazione ignota) “DONNA CHE NON VUOLE ESSERE NOMINATA” indicare il codice 999 per Cognome e Nome; non devono essere indicati il Codice sanitario, i comuni di nascita e di residenza; per la provincia di residenza indicare codice 999; indicare per esteso la provincia di nascita e solo l’anno per la data di nascita.

3. Nel caso di padre che non vuole essere nominato indicare codice 999 come nel caso di “DONNA CHE NON VUOLE ESSERE NOMINATA”.

4. Per nato vivo si intende il prodotto del concepimento che, al momento della nascita, respiri o dimostri qualsiasi altro segno di vita quali, il battito cardiaco o la pulsazione del cordone ombelicale o movimenti dei muscoli volontari, indipendentemente dalla durata della gestazione

5. Per nato morto si intende il prodotto del concepimento che, una volta espulso o completamente estratto dal corpo materno, non abbia respirato o manifestato alcun segno di vita (battito cardiaco o la pulsazione del cordone ombelicale o movimenti dei muscoli volontari), purché siano trascorsi almeno 180 giorni di amenorrea. NEL CASO DI NATO MORTO DEVE ESSERE COMPILATA LA SEZIONE D) DEL CEDAP; NON DEVE ESSERE COMPILATA LA SCHEDA DI MORTE ENTRO IL PRIMO ANNO DI VITA.

6. Per nato pretermine si intende qualsiasi nato vivo con meno di 37 settimane complete di età di gestazione (cioè nato entro il 259° giorno compiuto), calcolata a partire dal primo giorno dell’ultimo ciclo mestruale.

7. Per aborto spontaneo si intende il prodotto del concepimento che, una volta espulso o completamente estratto dal corpo materno, non abbia respirato o manifestato alcun segno di vita (battito cardiaco o la pulsazione del cordone ombelicale o movimenti dei muscoli volontari), purché l’evento si verifichi entro il 180° giorno di amenorrea.

8. Morto nel primo mese di vita (morte neonatale secondo la legge italiana) è un soggetto nato vivo (vedere nota 4) e deceduto entro il compimento del 30° giorno di vita dal momento della nascita.

Nel caso di morte neonatale e in generale nel caso di morte entro il compimento del 1° anno di vita (età inferiore a 365 giorni) deve essere compilata dal medico la SCHEDA DI MORTE ENTRO IL PRIMO ANNO DI VITA (mod. ISTAT/D4 e ISTAT/D5).

9. Per difetto di accrescimento fetale si intende il rallentato accrescimento intrauterino (valori inferiori al 10° percentile) diagnosticato in fase prenatale.

10. Nel caso il concepimento sia avvenuto attraverso l’utilizzo di tecniche di riproduzione medico-assistita specificare il metodo seguito:

1 - solo trattamento farmacologico per induzione dell’ovulazione;

2 - IUI (Intra Uterine Insemination);

3 - GIFT (Gamete Intra Fallopian Transfer);

4 - FIVET (Fertilization in Vitro and Embryo Transfer);

5 - ICSI (Intra Cytoplasmic Sperm Injection);

6 - altre tecniche.

11. “Luogo del parto”: se avvenuto in “istituto pubblico-privato” (1) oppure in “altra struttura” (3) indicare il codice dell’istituto e la denominazione; se avvenuto in “abitazione” (2) o in “altro” (4) indicare il comune e l’indirizzo.

12. ‘Modalità del travaglio’: per induzione del travaglio con farmaci si intende l’utilizzo di ossitocina, prostaglandine. Per travaglio pilotato si intende l’uso di farmaci durante il travaglio, magari insorto spontaneamente, per correggerne le devianze dalla normalità. Con la modalità “senza travaglio” si identificano i casi in cui si procede in elezione o d’urgenza ad un Taglio Cesareo senza che si sia avviato spontaneamente o sia stato indotto un travaglio di parto.

13. ‘Modalità del parto’: con “parto spontaneo” si delimita il concetto di spontaneità all’assenza di operatività vaginale; viceversa devono essere inclusi i parti in cui si pratichi l’episiotomia. Con ‘cesareo in elezione’ devono essere considerati anche i casi conseguenti ad una fallita induzione, che non sono una vera e propria elezione, ma sono prossimi a questa. Con ‘cesareo in travaglio’ devono essere considerati anche i casi di Taglio Cesareo in urgenza (es. placenta previa, distacco di placenta). Il parto cesareo deve essere registrato solo con i codici 2) cesareo in elezione o per fallita induzione e 3) cesareo in travaglio o in urgenza. Il ricorso al cesareo, conseguente a una richiesta della donna, valutata e accettata dal clinico deve essere registrata con codice 2).
Nel caso di parti plurimi la variabile ‘Modalità del parto’ può venire registrata per ogni singolo nato.

14. Nel caso di parto plurimo le notizie riguardanti i nati successivi al primo devono essere riportate nel MODULO AGGIUNTIVO PER I PARTI PLURIMI.

15. Non debbono essere ritenute “rianimazione” le comuni stimolazioni fisiche (le piccole percussioni del torace, sulle piante dei piedi, etc., la detersione del volto, etc.) le modeste supplementazioni di ossigeno somministrate per pochi secondi e, in generale, le manovre che rientrano nell’ambito delle cure minime riservate ai neonati con Apgar al primo minuto normale o lievemente inferiore a 7, ma con pronta ripresa e successiva gestione sovrapponibile a quella riservata ai neonati considerati senza problemi.

16. L’indicazione di sesso incerto ha valore ai fini conoscitivi e non altera le norme vigenti relative all’obbligo di dichiarazione del sesso alla nascita, né influisce sulla successiva eventuale modifica del sesso.

17. In presenza di nati vivi con malformazioni congenite viene compilata, da parte del medico accertatore, la sezione E del certificato, che sostituisce il “modello 51 sanità pubblica”, concernente la denuncia di nato con malformazioni congenite, quale strumento di base utile per la rilevazione dei dati essenziali (art. 3 del Decreto 16 luglio 2001, n. 349).

D) INFORMAZIONI SULLE CAUSE DI NATI-MORTALITÀ (codici ICD-9-CM) **_(5)_**:

malattia o condizione morbosa principale del feto :

altra malattia o condizione morbosa del feto :

malattia o condizione morbosa principale della madre interessante il feto :

altra malattia o condizione morbosa della madre interessante il feto :

altra circostanza rilevante :

momento della morte (barrare una sola voce):

1 prima del travaglio 3 durante il parto

2 durante il travaglio 4 sconosciuto

esecuzione esami strumentali in caso di malformazioni:

1 si 2 no

esecuzione fotografie in caso di malformazioni:

1 si 2 no

riscontro autoptico:

1 causa della morte confermata dall’autopsia

2 risultato dell’autopsia non ancora disponibile

3 autopsia non effettuata

E) INFORMAZIONI SULLA PRESENZA DI MALFORMAZIONI (solo per i nati vivi):

malformazioni diagnosticate (indicare massimo 3 malformazioni - codici ICD-9-CM):

1a m. :

2a m. :

3a m. :

cariotipo del nato (se effettuato prima della nascita):

età gestazionale alla diagnosi di malformazione (in settimane compiute): 99 Non rilevato

età neonatale alla diagnosi di malformazione (in giorni compiuti): 99 Non rilevato

eventuali malformazioni in famiglia (barrare una o più voci):

fratelli 1 si 2 no

madre 1 si 2 no

padre 1 si 2 no

genitori madre 1 si 2 no

genitori padre 1 si 2 no

altri parenti madre (fratelli/cugini/zii) 1 si 2 no

altri parenti padre (fratelli/cugini/zii) 1 si 2 no

malattie insorte in gravidanza (indicare massimo 2 malattie - codici ICD-9-CM):

1a m. :

2a m. :

data di compilazione

FIRMA DEL MEDICO
