# Supplementary material for: Length of stay following vaginal deliveries: A population based study in the Friuli Venezia Giulia region (North-Eastern Italy), 2005-2015
Source: PLoS One. 2019 Jan 3;14(1):e0204919. doi: 10.1371/journal.pone.0204919 (PMC6317786; doi:10.1371/journal.pone.0204919)
Supplement: S1 Table — Sensitivity model A: multiple logistic regression analysis adjusted for hospital and all other factors Including pre-term history and marital status. Sensitivity model B: multiple logistic regression analysis adjusted for hospital and all other factors, but pre-term history and marital status. Odds ratio (OR) with 95% confidence interval (95%CI); LoS = length of hospital stay; NA = not available; observations = complete (case analysis) observations. (DOCX) [file pone.0204919.s002.docx]

| **Table S2. Sensitivity Analysis.** Sensitivity model A: multiple logistic regression analysis adjusted for hospital and all other factors Including pre-term history and marital status. Sensitivity model B: multiple logistic regression analysis adjusted for hospital and all other factors, but pre-term history and marital status. Odds ratio (OR) with 95% confidence interval (95%CI); LoS= length of hospital stay; NA= not available; observations= complete (case analysis) observations | | | | | |
| --- | --- | --- | --- | --- | --- |
| **FACTORS** | **STRATA** | **VAGINAL DELIVERY MODE** | | | |
|  |  | **SPONTANEOUS**  **(LoS >2 vs. ≤ 2 days)** | | **INSTRUMENTAL**  **(LoS >3 days vs. ≤ 3 days)** | |
|  |  | **Sensitivity**  **Model A**  (67,352 observatons) | **Sensitivity**  **Model B**  (73,281 observations) | **Sensitivity**  **Model A**  (6,521 observations) | **Sensitivity**  **Model B**  (7,050 observations) |
| **HOSPITAL** | **A** | reference | reference | reference | reference |
|  | **B** | 80.19 (69.89; 91.99) | 89.37 (78.49; 101.77) | 7.91 (6.34; 9.86) | 7.92 (6.39; 9.80) |
|  | **C** | 4.80 (4.45; 5.18) | 4.86 (4.51; 5.23) | 0.82 (0.59; 1.16) | 0.83 (0.59; 1.17) |
|  | **D** | 25.86 (21.56; 31.02) | 26.47 (22.35; 31.35) | 9.46 (5.86; 15.28) | 7.86 (5.09; 12.13) |
|  | **E** | 7.68 (7.00; 8.42) | 8.40 (7.68; 9.19) | 1.97 (1.46; 2.65) | 2.22 (1.67; 2.94) |
|  | **F** | 3.08 (2.82; 3.37) | 2.93 (2.69; 3.20) | 0.83 (0.61; 1.14) | 0.79 (0.58; 1.08) |
|  | **G** | 0.73 (0.68; 0.79) | 0.77 (0.72; 0.83) | 0.76 (0.58; 1.00) | 0.73 (0.56; 0.94) |
|  | **H** | 2.81 (2.64; 3.00) | 2.78 (2.61; 2.96) | 1.51 (1.18; 1.92) | 1.53 (1.21; 1.94) |
|  | **I** | 9.41 (8.46; 10.47) | 10.42 (9.49; 11.43) | 2.76 (2.00; 3.81) | 2.85 (2.15; 3.78) |
|  | **J** | 2.34 (2.19; 2.50) | 2.39 (2.24; 2.55) | 2.51 (1.98; 3.17) | 2.56 (2.03; 3.24) |
|  | **K** | 10.13 (9.28; 11.04) | 10.29 (9.45; 11.20) | 2.33 (1.80; 3.01) | 2.41 (1.88; 3.10) |
|  | **L** | NA | NA | NA | NA |
| **Calendar year (2005-2015)** | | 0.96 (0.95; 0.97) | 0.96 (0.95; 0.96) | 0.98 (0.96; 1.00) | 0.97 (0.95; 0.99) |
